# *Wolbachia* Age-Sex-Specific Density in *Aedes albopictus*: A Host Evolutionary Response to Cytoplasmic Incompatibility?

**DOI:** 10.1371/journal.pone.0009700

**Published:** 2010-03-16

**Authors:** Pablo Tortosa, Sylvain Charlat, Pierrick Labbé, Jean-Sébastien Dehecq, Hélène Barré, Mylène Weill

**Affiliations:** 1 Institut des Sciences de l'Evolution, CNRS UMR 5554, Université Montpellier 2, Montpellier, France; 2 Centre de Recherche et de Veille sur les Maladies Emergentes dans l'Océan Indien, Ste Clotilde, France; 3 Laboratoire de Biométrie et Biologie Evolutive, CNRS UMR 5558, Université Claude Bernard Lyon 1, Villeurbanne, France; 4 Direction Régionale des Affaires Sanitaires et Sociales de La Réunion, Saint-Denis, France; 5 Laboratoire Parasites et Ecosystèmes Méditerranéens, Université de Corse, Corte, France; Queens University, Canada

## Abstract

**Background:**

*Wolbachia* bacteria have invaded many arthropod species by inducing Cytoplasmic Incompatibility (CI). These symbionts represent fascinating objects of study for evolutionary biologists, but also powerful potential biocontrol agents. Here, we assess the density dynamics of *Wolbachia* infections in males and females of the mosquito *Aedes albopitcus*, an important vector of human pathogens, and interpret the results within an evolutionary framework.

**Methodology/Principal Findings:**

*Wolbachia* densities were measured in natural populations and in age controlled mosquitoes using quantitative PCR. We show that the density dynamics of the *w*AlbA *Wolbachia* strain infecting *Aedes albopictus* drastically differ between males and females, with a very rapid decay of infection in males only.

**Conclusions/Significance:**

Theory predicts that *Wolbachia* and its hosts should cooperate to improve the transmission of infection to offspring, because only infected eggs are protected from the effects of CI. However, incompatible matings effectively lower the fertility of infected males, so that selection acting on the host genome should tend to reduce the expression of CI in males, for example, by reducing infection density in males before sexual maturation. The rapid decay of one *Wolbachia* infection in *Aedes albopictus* males, but not in females, is consistent with this prediction. We suggest that the commonly observed reduction in CI intensity with male age reflects a similar evolutionary process. Our results also highlight the importance of monitoring infection density dynamics in both males and females to assess the efficiency of *Wolbachia*-based control strategies.

## Introduction

*Wolbachia* are maternally inherited bacteria that optimize their own fitness by manipulating the reproduction of their host [1]. Among the reported manipulations, Cytoplasmic Incompatibility (CI) is found in several mosquito species and is by far the best documented. In the simplest CI picture, uninfected females' embryos rapidly die following fertilization by *Wolbachia* infected males' sperm. In contrast, infected females' eggs are “immune” to this lethal effect and develop normally into infected adults, so that the infection frequency tends to increase. Similar embryonic mortality is seen in crosses between males and females harbouring different, incompatible, *Wolbachia* variants [2].

This pattern produces two consequences of interest for the present study. First, it makes *Wolbachia* a very promising tool for pest species control strategies: *Wolbachia* can be used as a sterilizing factor when present in males, and is also a potential powerful vector to drive transgenes through host populations [3]. Second, the drastically different effects of infection on males and females have interesting evolutionary consequences that should be taken into consideration when envisaging *Wolbachia*-based control strategies. *Wolbachia* acts as a rescue factor when present in infected females' embryos; for this reason, the bacteria and its host's genome are expected to cooperate and optimize transmission to offspring [4]. However, the lethal effect of infected males' sperm expressed in crosses with incompatible females is detrimental to the transmission of the host nuclear genes. For this reason, theory predicts that selection acting on host nuclear genes should tend to prevent this effect, for example by reducing the density of infection in males before they become sexually mature [4], [5].

In the present work, we use natural populations and age-controlled specimens to show that one *Wolbachia* strain infecting the mosquito *Aedes albopictus* follows the above-predicted density dynamics. We show that females are always co-infected with *Wolbachia* strains *w*AlbA and *w*AlbB, with bacterial densities slightly increasing with aging. Males display a drastically different pattern: they are co-infected at emergence but *w*AlbA density quickly decreases towards complete loss with male aging. In addition, we investigate the putative role of a WO bacteriophage, previously described in *Ae. albopictus*, in this pattern. WO can alternate lytic and lysogenic cycles and may therefore control *Wolbachia* densities as shown for the parasitoid wasp *Nasonnia vitripennis* [6]. Our data show that WO density is driven by *w*AlbB and not *w*AlbA, confirming previously published data [7] and suggesting that the involvement of this phage in the *w*AlbA depletion is unlikely.

*Ae. albopictus* is raising much concern after multiple invasive episodes documented over the last 20 years [8] with serious medical consequences such as the recent Chikungunya epidemics in the Indian Ocean [9] and Italy [10]. Any *Wolbachia*-based control strategy targeting this species would require the introduction of a new bacterial strain incompatible with the resident infections. Our results emphasize that such strategies should take into consideration the possibility of sex-dependent *Wolbachia* dynamics, which could impede the expression of CI.

## Results

### Sex-specific* infection patterns*

Infection status of wild male and female *Ae. albopictus* collected on La Réunion Island was determined by a standard PCR assay [11]. Surprisingly, all females were found bi-infected while roughly half of the males were singly infected with *w*AlbB (Table 1). The prevalence of the *w*AlbA infection differed significantly between males and females (Fisher's exact test, *p*<0.001). Mosquitoes collected on Madagascar were also genotyped following this procedure and displayed the same sex-specific infection pattern although sample size was too small to give significant results (Fisher's exact test, *p* = 0.1, Table 1).

**Table 1:** Infection status of wild caught *Ae. albopictus* as determined with standard PCR [11].

Collection site	Sex	N	A−B+	A+B+	*p*-value
**La Réunion Island**	Males	39	20	19	
	Females	40	0	40	*<0.001*
**Madagascar**	Males	3	3	0	
	Females	2	0	2	0.1

A−B+ and A+B+ refer to *w*AlbB singly infected and *w*AlbA/*w*AlbB co-infected mosquitoes, respectively. Differences between male and female infection patterns were analysed using Fisher's exact test (*p*-values are reported for each sample, in italics when significant).

We used a recently developed quantitative PCR approach [7] to further investigate this result. This alternative method pointed out that most of the specimens previously characterised as *w*AlbB mono-infected males were actually co-infected with scarce densities of *w*AlbA, undetectable by standard PCR. In addition, quantitative PCR revealed highly variable *w*AlbB/*w*AlbA ratios in males, ranging from 1 to 5000. The previously described PCR [11] and q-PCR [12] protocols use a common downstream primer annealing on *w*AlbA and *w*AlbB *wsp* sequences (*i.e.* primer 691R) which we suspect may have been titrated by the overwhelming *w*AlbB bacteria, leading to the observed false negative results. This hypothesis was substantiated by a standard PCR assay using *w*AlbA-*wsp* specific primers [7] which revealed that 4 out of 5 specimens characterised as negative for *w*AlbA with the previously described PCR primers [11] were actually positive.

**Figure 1:**
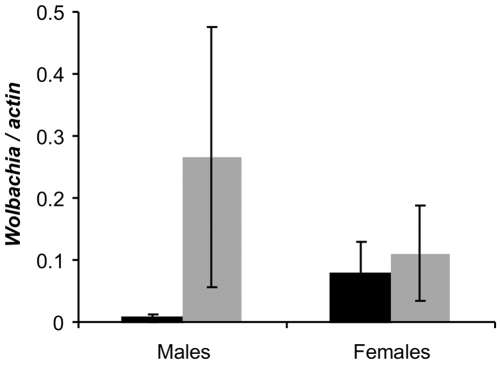
Mean *Wolbachia* density in wild specimens from La Réunion Island. Each DNA was quantified in triplicate and the average density was calculated for each specimen. Mean density in the population is reported for each sex and is depicted in black for *w*AlbA and grey for *w*AlbB. Standard error is calculated on the mean of all average densities (males N = 15, females N = 10).

Quantification data (Fig. 1) showed that densities of both *w*AlbA and *w*AlbB *Wolbachia* differed significantly among males and females (Generalized Linear Model (GLM): *density* = *sex*, where *density*, the response variable, is the result of the qPCR analyses, square-root transformed to ensure the normality of the residuals, and *sex* a factorial explanatory variable with two levels, male or female: *F* = 57.9, *P*<0.001 for *w*AlbA, *F* = 6.3 and *P* = 0.019 for *w*AlbB). This approach thus confirmed that *Wolbachia* density pattern is strongly dependent upon sex in *Ae. albopictus*.

### Age and sex-specific infection patterns

**Figure 2:**
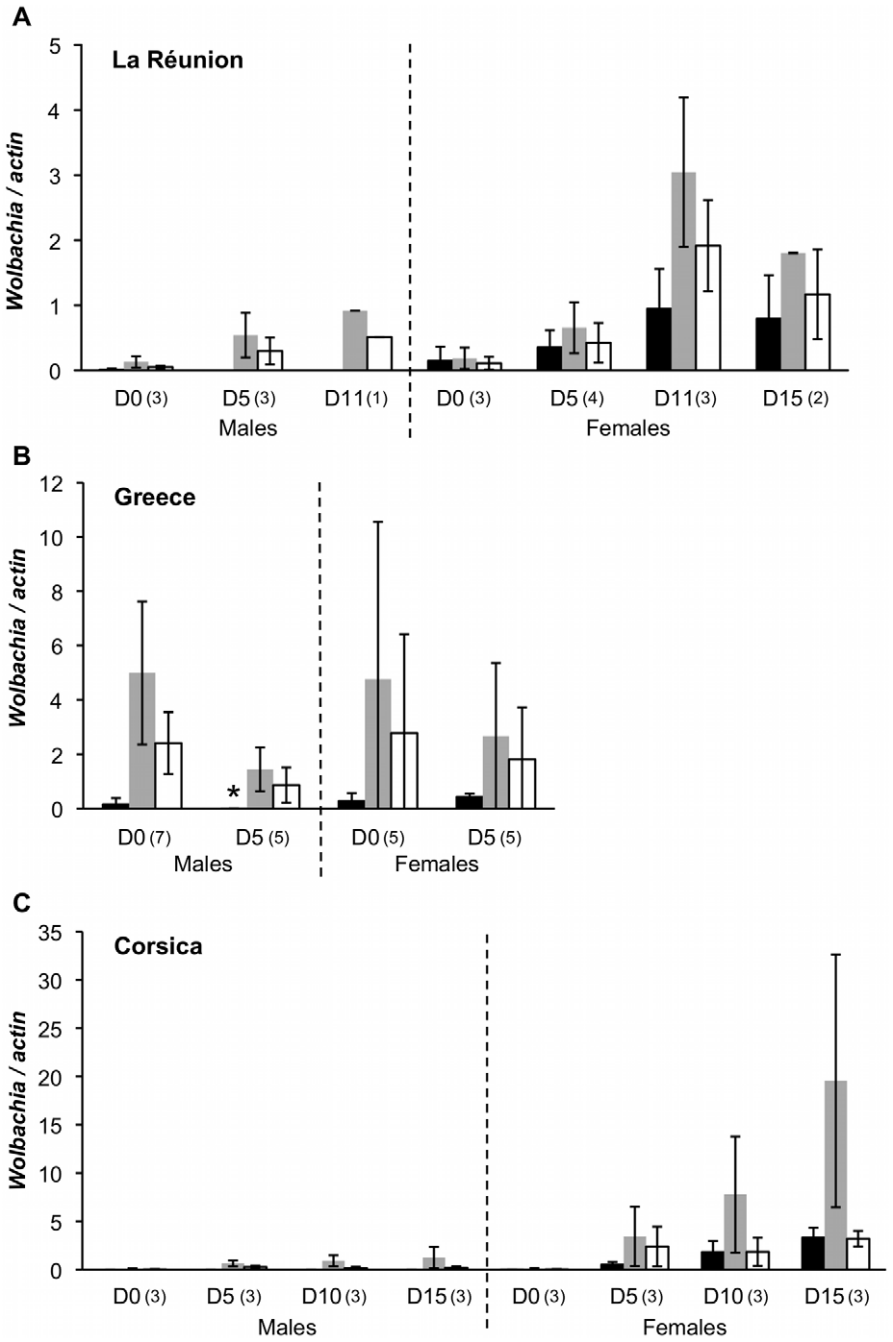
Effect of aging on *Wolbachia* and WO prophage densities. The age is indicated on the x axis as days (D) after emergence, for males and females separately. Plots report average *w*AlbA (black), *w*AlbB (grey) and WO prophage (white) density for each age and sex in samples from (A) La Réunion Island, (B) Greece (* the very low but non-zero density seen in 5-days-old males from Greece is not visible on the figure (mean = 3.4×10^−3^; standard error = 4.2×10^−3^)), and (C) Corsica. Numbers into brackets indicate the number of individuals analysed.

All wild females tested were co-infected. This pattern suggests a highly efficient vertical transmission of both infections, making it unlikely that singly infected males received only the *w*AlbB strain from their mother. In addition, should singly infected embryos be produced, we expect most of those to be eliminated through CI. We therefore conjectured that *w*AlbA may be lost after fertilization specifically in male individuals and tested this hypothesis. Eggs were obtained with an oviposition trap deposited on La Réunion Island from the site where the wild larvae had been previously collected. Eggs were dried for 2 days at room temperature and hatching was subsequently induced by immersion into water. Larvae were reared until emergence and adult specimens from each sex were tested at different ages (0, 5, 11 and 15 days after emergence). Total DNA was prepared and analysed by qPCR. The results, reported on figure 2A, show that all females were co-infected. The pattern was drastically different in males, with *w*AlbA densities being very low at emergence and decreasing with aging until no *w*AlbA could be detected within 5 days after emergence.

Two similar experiments were carried out using mosquitoes collected in Greece and Corsica. Mosquitoes were reared under laboratory conditions and individually tested at different times after emergence. The results showed that the above described age-sex-specific pattern is geographically widespread, although some quantitative variation is seen. In the Greek lines, both *w*AlbA and *w*AlbB were detected in all tested females and males, but old males exhibited scarce levels of *w*AlbA (Fig 2B). For mosquitoes from Corsica, the pattern turned out to be very sharp: all tested males were devoid of *w*AlbA, regardless of age, suggesting the *w*AlbA decrease might take place earlier or faster in this line (Fig. 2C). *Wolbachia w*AlbA and *w*AlbB densities were variable depending on the mosquitoes origin, a result previously described in the field [13].

Data from these three quantitative experiments were analyzed together using a GLM in order to simultaneously explore the effects on *Wolbachia* density of three different variables (*age*, *sex* and *population*), acting either independently or in interaction. In a first model taking *w*AlbA as the variable of interest, *Wolbachia* density is found significantly affected by *age* in interaction with both the *sex* and *population* factors (*F* = 17.8 and *P*<0.001; *F* = 8.64 and *P*<0.001, respectively). Interestingly, similar results are obtained in a second model, taking *w*AlbB as the variable of interest (*sex* and *age*: *F* = 5.13 and *P* = 0.001; *sex* and *population*: *F* = 9.7 and *P*<0.001). This analysis thus confirms that the density of *w*AlbA is affected by age and sex in all populations, but further suggests that these variables also affect the *w*AlbB density.

To further investigate the effect of age and sex on each infection, a direct correlation between density and age was investigated in both sexes by using Spearman correlation tests. Three independent tests corresponding to each population were run sequentially. Multiple testing was taken into account using the Bonferroni sequential procedure according to Hochberg [14]. The *w*AlbA density was significantly positively correlated with age in females from La Réunion Island and Corsica (*ρ* = 0.637 and *P* = 0.026; *ρ* = 0.93 and *P*<0.001, respectively) but not in females from Greece. In contrast, the *w*AlbA density was negatively correlated with age in males from La Réunion Island and Greece (*ρ* = −0.89 and *P* = 0.007; *ρ*  = −0.76 and *P* = 0.004, respectively). The test could not be implemented with the Corsican males which are not infected by *w*AlbA. For *w*AlbB density, results were generally less clear: a significant positive correlation was found between age and *w*AlbB density in females from La Réunion Island (*ρ* = 0.86 and *P*<0.001) and Corsica (*ρ* = 0.91 and *P*<0.001), but none for females from Greece; a positive correlation was found in males from Corsica (*ρ* = 0.69 and *P* = 0.013) and from La Réunion Island (*ρ* = 0.85 and *P* = 0.016; not significant after Bonferroni correction), but the correlation was negative in males from Greece (*ρ* = −0.81 and *P* = 0.001).

Overall, our data show that both *Wolbachia* types increase with aging in females. In contrast, in males, the *w*AlbA density strongly decreases with age, towards complete loss in males from La Réunion Island while males from Corsica are depleted of this *Wolbachia* at emergence. *w*AlbB density correlates either positively or negatively with age depending on the source sample.

### Assessing WO phage involvement in infection patterns

The density of the WO phage previously described in *Ae. albopitcus* was measured in the same mosquitoes by qPCR (Fig. 2) as previously described [7]. The involvement of this phage in the observed pattern was investigated by computing data from the three quantitative experiments in a GLM. This analysis allowed exploring simultaneously the effects of *w*AlbA and *w*AlbB densities on WO density, while controlling for the variables *age*, *sex* and *population*. The model was log(WO) = sex*age*log(*w*AlbB) + sex*age*log(*w*AlbA) + *population*, where WO, *w*AlbA and *w*AlbB are the quantification of the corresponding element (quantitative variables); the values where log-transformed to ensure normality of the residuals; * indicates that the model considers both the main and interaction effects of the variables. We found a significant effect of *age*:*w*AlbB interaction (*F* = 4.55, *P* = 0.037), a significant effect of population (*F* = 3.58, *P* = 0.034) and a highly significant effect of *w*AlbB (*P*<0.001), while all other interactions or main effects were non significant (*P*>0.05). This analysis thus confirms that the density of this phage is driven only by the density of *w*AlbB, as shown in a previous study [7]. From these results, we can conclude that the WO phage previously described in *Ae. albopitcus* is not responsible for the decrease of *w*AlbA density in aging males.

## Discussion

CI causes the death of embryos in crosses between males carrying a given *Wolbachia* strain and females devoid of a compatible infection [15]. Females' offspring infected by a compatible *Wolbachia* are immune to this effect so that the infection frequency tends to increase [2]. The presence of *Wolbachia* is crucial to the survival of infected females' offspring, as only these bacteria can restore the functionality of the sperm “modified” by the paternal *Wolbachia*. Therefore, selection acting on the host nuclear genome is expected to optimise the efficiency of *Wolbachia* maternal transmission [4], [16]. Selection on hosts and symbionts should thus converge toward improved transmission. However, if incompatible crosses do occur, then infected males suffer a fertility reduction. In other words, the expression of CI in a male can be detrimental to the transmission of its nuclear genes, so that selection is expected to suppress the expression of CI, for example by eliminating the infection during development [4], [5]. This is the exact pattern we observe for the *w*AlbA infection in *Ae. albopictus*. Male embryos receive the *w*AlbA infection from their doubly infected mothers, and are therefore immune to CI-induced embryonic mortality, but they lose this infection as they become older (Fig. 2). Although other interpretations can be envisaged, including non-adaptive ones, it is tempting to propose that the loss of *w*AlbA in adult *Ae. albopictus* males ultimately results from an evolutionary process selecting nuclear counter-measures to *Wolbachia* manipulation. The decrease of *w*AlbA in males might in turn produce a decline of *w*AlbA-induced CI, which is an assumption of this rational. Notably, age has been found to affect the expression of CI in several systems, sometimes in relation with *Wolbachia* density in testes [17], [18], [19], [20], [21], [22]. In particular, a marked decrease in CI was previously observed in 10-days-old *w*AlbA mono-infected *Ae. albopictus* males grown in laboratory conditions [23]. The decrease of *w*AlbA densities with aging observed in the present work may be the primary cause of this phenomenon. However, this hypothesis remains to be thoroughly tested since decrease in *Wolbachia* density is not always associated with CI reduction [24], [25]. Furthermore, the low *w*AlbA densities quantified here in DNA extracts from entire mosquito bodies might not correlate with densities in testes specifically [26].

Although double infection is virtually fixed among females in modern populations [27], suggesting that the loss of *w*AlbA in males is hardly adaptive nowadays, it is plausible that it was adaptive in the recent past, when the *w*AlbA infection was not fixed. Besides, rare events of *w*AlbA loss in females through imperfect transmission could produce a strong enough pressure to select for the loss of *w*AlbA in adult males.

The *w*AlbA and *w*AlbB infections show markedly different density patterns with regard to age and sex. Under the hypothesis that the decrease of *w*AlbA density in males is adaptive, the non-decrease of *w*AlbB can appear puzzling. The following hypotheses can be proposed to account for this contrast. A first possibility is that the spread of *w*AlbB in *Ae. albopictus* is a very recent event, consistent with the observation that two lab-maintained strains, collected in Mauritius and Samui Islands before 1970, carry only *w*AlbA [27]. A second plausible explanation would relate to differences in the vertical transmission efficiencies of the two strains. If *w*AlbB is more efficiently transmitted from females to their offspring than *w*AlbA, then *w*AlbB infected males suffer less fertility reduction due to CI. In such a case, the selective pressure for a reduction of density in males would be reduced for *w*AlbB. The first hypothesis could be tested using museum specimens, collected in the past, an approach used with success in other systems [28]. Testing the second hypothesis would require accurate measures of transmission efficiencies, controlling for the elimination of uninfected offspring through CI.

In addition to the above, potential interactions between *w*AlbA, *w*AlbB and the host genome would be worth investigating. Indeed, the *w*AlbA depletion might rely in part on the presence of the *w*AlbB strain, through competitive interactions. However, the previous observation that CI expression reduces with aging in *w*AlbA mono-infected males suggests that such interaction cannot be the full explanation for the *w*AlbA depletion. The comparison of *w*AlbA dynamics in mono-infected vs. bi-infected males, in a controlled genomic background, would allow addressing this issue.

We carried out quantitative PCR to test whether the dramatic decline of *w*AlbA in males is caused by entrance in lytic cycle of a WO phage previously described in *Ae. albopictus*. Our data show that the density of this phage does not increase in males and is driven by *w*AlbB and not *w*AlbA in all three populations. This result confirms similar data obtained on a mosquito population from La Réunion [7], [25], and further suggests that the WO-*w*AlbB linkage is not restricted to mosquitoes from La Réunion but appears to be geographically widespread. These data further suggest that this phage is not involved in the *w*AlbA disappearance observed specifically in aging males. Notably, our detection method specifically targets the previously described WO phage from *Ae. albopictus*. Hence the possibility remains that other WO variants, possibly integrated in *w*AlbA, might be involved in the observed pattern.

The use of *Wolbachia* in alternative, environment-friendly vector control strategies is currently investigated in several laboratories [29], [30]. CI can be used for mass production of sterilizing males in a derivative of standard sterile insect techniques [31]. The present work emphasizes a problem potentially associated with *Wolbachia*-based vector control strategies. The possibility of *Wolbachia* loss associated with CI decrease in aged males needs to be investigated since it may be detrimental to sterilization strategy. Interestingly, it has been recently reported that *Ae albopictus* males in La Réunion show an unexpectedly high mean life expectancy, ranging from 16.2 to 24.5 days [32], which would strengthen the impact of density reduction on the effective expression of CI in the field. Therefore, any attempt of producing incompatible males should include CI monitoring over the whole male lifespan. If not appropriately controlled, sterilizing properties of the released males may decrease with aging and therefore compromise the success of such an appealing strategy.

## Methods

### Mosquito rearing

*Wolbachia* genotyping in *Ae. albopictus* natural populations was carried out on mosquitoes collected on La Réunion Island and Madagascar. Mosquitoes from La Réunion Island were collected in the field as larvae, brought to adulthood in the laboratory and immersed into ethanol after emergence. The 5 specimens from Madagascar were collected as adults and directly immersed into ethanol.

Age controlled experiments were carried out as follows: *Ae. albopictus* eggs were collected on Réunion Is. and Corfu Is. with ovitraps made of black-painted soda cans containing water and ovipositing brown paper. Corsican mosquitoes eggs derived from a first generation reproduced in laboratory.

Filter papers were dried for 48h after oviposition and subsequently immersed into fresh water to induce hatching. When larvae reached pupal stage, each bowl was placed inside a mosquito cage (20cm×20cm×20cm). After emergence, adults were maintained at room temperature with daylight exposure and fed with cotton soaked in honey. Adult specimens were eventually frozen and stored in ethanol.

### *Wolbachia* genotyping

Total DNA was extracted using a hexadecyltrimethylammonium bromide (CTAB) protocol [33]. Infection status was determined following a previously described method allowing specific amplification of either *Wolbachia* A (81F and 691R primers) or *Wolbachia* B (183F and 691R primers) clades [11]. In addition, the same PCR conditions were used to specifically amplify *w*AlbA-*wsp* locus using the QAdir1 and QArev2 primers, previously developed for qPCR assays [7].

### Quantitative (q)PCR

*w*AlbA, *w*AlbB and WO prophage were quantified in *Ae. albopictus* following a previously described method [7]. About 2ng of genomic DNA was mixed with primers (0.5 µM) amplifying specifically either *w*AlbA, *w*AlbB, WO or *act* locus, 2 µl of anti-*Taq*-containing master mix and complemented to 20 µl with water. Master mix and anti-*Taq* antibody were used according to Roche LightCycler instructions for SYBR technology [34]. PCR was run for 45 cycles (94°C for 4 s, 65°C for 14 s, and 72°C for 19 s). The pQuantAlb plasmid, containing a single copy of *w*AlbA, *w*AlbB, WO and *actin* template from *Ae. albopictus* nuclear DNA, was serially diluted to build a standard curve with all four *loci* present at an equimolar concentration. Thus same dilutions were amplified with the four specific couples in each qPCR run so that signals could be easily standardized with the nuclear *actin* reference. For each mosquito, quantification measurements were triplicated and mean genome number of *w*AlbA, *w*AlbB and WO was obtained per mosquito nuclear *actin* copy number.

### Statistical analysis

All statistical analyses were computed using the free software R (http://www.r-project.org/) using methods, tests and model simplification procedures as described in Crawley [35].
